# Differential Organ Ageing Is Associated With Age‐Related Macular Degeneration

**DOI:** 10.1111/acel.14473

**Published:** 2025-01-05

**Authors:** Anastasios Papadam, Arimantas Lionikas, Felix Grassmann

**Affiliations:** ^1^ Institute of Medical Sciences, University of Aberdeen Aberdeen UK; ^2^ Institute for Clinical Research and Systems Medicine Health and Medical University Potsdam Germany

**Keywords:** ageing, age‐related macular degeneration, biological age, genetics, machine learning

## Abstract

Age‐related macular degeneration (AMD) is a progressive disorder and the leading cause of central vision loss. Age is the most important risk factor, followed by genetics and smoking. However, ageing is a complex process, and biological age can deviate from chronological age between individuals and within different organ systems. Initially, we used machine learning to predict the biological age of the immune, cardiovascular, pulmonary, renal, musculoskeletal, metabolic and hepatic systems by analysing various physiological and physical markers in the UK Biobank cohort. Then, we investigated the association of each organ's biological age with incident AMD derived from electronic health record data as well as with different AMD genetic risk scores. We observed that most organ systems in participants who developed AMD after recruitment showed accelerated ageing compared with controls, with the immune system being the most affected, especially in younger males. Surprisingly, we found that AMD patients showed slower ageing of their hepatic system compared to controls, particularly in female patients. The overall AMD genetic risk score was associated with faster organ ageing across all tissues except cardiovascular and pulmonary, while genetic risk scores stratified by pathways differently influenced each organ system. In conclusion, we found differential organ ageing associated with AMD. Significantly, genetic risk variants of AMD are associated with differential ageing of various organ systems.

## Graphical abstract

Using the UK Biobank, this study trained neural networks to estimate the biological age of individuals according to markers of organ function. Computing the difference between the estimated organ age and chronological age yielded an estimate for accelerated or slowed ageing. The age difference was then used to assess whether individuals with incident age‐related macular degeneration have faster or slower organ ageing compared to controls.

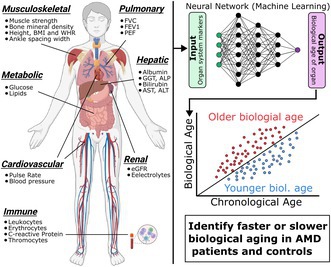

## 1 Introduction

Age‐related macular degeneration (AMD) is a complex progressive disease affecting the macula, the central part of the retina. It is the main cause of irreversible loss of central vision in individuals above 60 years old in Western societies (Wong et al. 2014). Currently, over 200 million people across the globe suffer from AMD, and the number of AMD patients is expected to increase by 88 million within the next decade (Wong et al. 2014).

The disease's hallmark is the accumulation of small yellow fat deposits between the retinal pigment epithelium (RPE) and the choroid, called drusen, accompanied by progressive loss of photoreceptor cells and RPE function. Late‐stage AMD is the most severe form and can manifest into Geographic Atrophy (GA) and/or Neovascular AMD (CNV). Currently, the progression of the neovascular form of AMD can be managed with anti‐angiogenic medication. However, the underlying disease associated with progressive loss of RPE and photoreceptors remains uncurable.

Over the past decade, extensive research has identified several risk factors for AMD. The most common risk factor is age, with genetics, smoking and BMI also being important contributors (Grassmann, Fauser, and Weber 2015). Gender may be a risk factor for AMD, but the scientific community still debates this (Winkler et al. 2018). In 2016, the International AMD Genomics Consortium (IAMDGC) conducted a large genome‐wide association study that validated and discovered new variants that are associated with AMD, increasing the total variants to 52 independent variants in 34 genomic loci (Fritsche et al. 2016; Grassmann, Heid, and Weber 2017a; Winkler et al. 2018). Newer studies based on meta‐analysing different cohorts have identified additional risk variants with ever‐decreasing effect sizes. The main pathways identified from those association studies point towards the role of the complement system, extracellular matrix homeostasis and lipid metabolism in the risk of the disease, explaining around half of the heritability of the disease (Fritsche et al. 2016).

The importance of chronological age as a risk factor for complex chronic diseases is well‐established (Niccoli and Partridge 2012). However, ageing is a complicated biological process that slowly weakens the efficiency and adaptiveness of cells, tissues and organs (Kennedy et al. 2014). Moreover, ageing trajectories can be different among individuals, as well as different organ systems of the same individual (Tuttle et al. 2020). An effective measurement of biological age should reflect potential differences in ageing trajectories between systems (Tuttle et al. 2020). Multiple models have been proposed to predict biological age, ranging from single biomarkers to large, advanced algorithms utilising various biomarkers involved in cellular, molecular and physiological processes (Ferrucci et al. 2020; Tian et al. 2023). In recent years, high‐throughput arrays measuring DNA methylation have allowed the capture of epigenetic variations. Those changes, also called epigenetic clocks (Hannum et al. 2013; Koch and Wagner 2011; Weidner et al. 2014), have been proven to be good predictors of biological age and are frequently found to be associated with diseases and mortality (Marioni et al. 2015). Thus, biological age can be a more insightful indicator of ageing and age‐related diseases compared to chronological age since it uses a variety of measurements related to the efficiency and adaptiveness of different organ systems (Ferrucci et al. 2020; Khan, Singer, and Vaughan 2017).

Therefore, we aimed to unveil the association between organ ageing and age‐related macular degeneration utilising physiological, blood‐bound, biometric biomarkers and known risk variants of epigenetic clocks available for multiple systems for the UK Biobank cohort (Graphical Abstract).

## 2 Methods

### 2.1 UK Biobank Description

The UK Biobank is a clinical thesaurus with genotypic and phenotypic information for 502,463 participants between the ages of 37 and 73 from Scotland, England and Wales (Sudlow et al. 2015). Most participants were genotyped and assessed through a questionnaire about their lifestyle and health between 2006 and 2010. Since then, the UK Biobank has been enriching its phenotypic dataset with health information for its participants using the UK national registers. The genetic information of the UK Biobank participants was obtained using Affymetrix BiLEVE and the UK Biobank Axiom arrays. The Affymetrix BiLEVE array was used for 49,950 participants, while the UK Biobank Axiom array was used for the rest. Both arrays have 784,256 autosomal variants and more than 95% overlap. We excluded participants who withdrew their consent, participants of non‐European ancestry, participants with sex chromosome abnormalities, participants who failed genotyping quality control and a low call rate (< 97%), resulting in 406,887 individuals (Figure S1). Body mass index (BMI) in the UK Biobank was ascertained at recruitment, and alcohol consumption and packyears smoked were ascertained from a questionnaire. The missing percentage of packyears ranged between 14.1% and 16.4% for the different AMD groups. Similarly, the percentage of individuals with missing packyear information ranged between 14.5% and 15.7% in our training and testing datasets. We set pack years in never‐smokers to zero. Current or past smokers with missing packyears were imputed to the respective median packyears in each group. Alcohol consumption was coded as 0 (less than three drinks a week) and 1 (more than three times a week). This corresponds to levels recently found to be indicative of the minimum‐risk drinking frequency in individuals who regularly drink alcohol (Hartz et al. 2018). Individuals who reported that they stopped drinking were also set to missing as they may have stopped drinking due to health issues. Missing values were then assigned as a separate level (‘missing’) in the association testing as they were not reported.

### 2.2 Physical and Physiological Markers

The UK Biobank offers a variety of physical and physiological measurements ascertained at recruitment representing several body systems. Each marker represents the efficiency and robustness of a specific body system (Graphical Abstract). The combination of these markers reveals the general condition of each body system:

1. Cardiovascular: Pulse rate, diastolic blood pressure, systolic blood pressure.
2. Pulmonary: Forced expiratory volume in 1 s, forced vital capacity, peak expiratory flow.
3. Musculoskeletal: Body Mass Index (BMI), height, weight, waist circumference, hip circumference, waist‐to‐hip ratio, phosphatase, calcium, alkaline phosphatase, vitamin D, hand grip left and right sides, heel bone mineral density and ankle space width.
4. Metabolic: Apolipoprotein A & B, cholesterol, glucose, glycated haemoglobin, lipoprotein A, triglycerides, HDL, LDL, LDL direct.
5. Immune: C reactive protein, WBC, RBC, haematocrit, mean platelet thrombocyte volume, Haemoglobin concentration, mean corpuscular haemoglobin concentration, red blood cell erythrocyte distribution width, platelet count, platelet crit, platelet distribution width, lymphocyte count, monocyte count, neutrophil count, eosinophil count, basophil count, nucleated red blood cell count, lymphocyte percentage, monocyte percentage, neutrophil percentage, eosinophil percentage, basophil percentage, nucleated red blood cell percentage, reticulocyte percentage, reticulocyte count, mean reticulocyte volume, mean sphered cell volume, immature reticulocyte fraction, high light scatter reticulocyte percentage, high light scatter reticulocyte count, mean corpuscular volume, mean corpuscular haemoglobin, direct bilirubin, total bilirubin, glycated haemoglobin HbA1c.
6. Renal: Creatinine enzymatic in urine, potassium in urine, sodium in urine, albumin, urea, urate, creatinine, cystatin, phosphate, total protein, alkaline phosphatase.
7. Hepatic: Alanine aminotransferase, aspartate aminotransferase, gamma‐glutamyl transferase, direct bilirubin, total bilirubin, total protein, albumin, alkaline phosphatase.
8. Epigenetic clocks: Genetic risk scores for intrinsic epigenetic age acceleration (IEAA), PhenoAge and HannumAge epigenetic clocks.

We imputed missing measurements in R (R Development Core Team 2000) using the R package ‘MICE’ and the maximum likelihood estimator (MLE). Measurements with more than 20% missingness were not included in the analysis. We imputed each system separately to prevent the potential ageing of one organ system from influencing the imputation of values belonging to other systems. Moreover, we included sex, smoking, BMI and chronological age for more accurate imputation. Using training data without imputation resulted in a similar performance; however, the number of missing predictions varied across the organ system, so we opted to impute the values instead.

### 2.3 AMD Participants in the UK Biobank

Previous studies defined AMD status using hospital record data or self‐reported data. Here, we present the ascertainment of AMD from the general practitioner (GP) dataset in the UK Biobank. Instead of ICD or other disease codes, the GP dataset contains so‐called read codes. Read codes are a vocabulary for clinicians to record patient findings, treatments and procedures used in the UK. We identified 14 read2 codes and 34 read3 codes (Table S1) corresponding to senile or age‐related macular degeneration in the documentation. Since there is no direct way to identify codes related only to age‐related macular degeneration, we pooled codes that shared the first 2 or 3 digits and assessed their association between known modifiable and non‐modifiable AMD risk factors. Specifically, we assessed their association with age, sex, smoking, *CFH* variant rs1061170, *ARMS2* variant rs10490924, and a genetic risk score summarising the risk of most AMD risk variants. We removed code families that failed to reach statistical significance with known modifiable or non‐modifiable AMD risk factors and also showed low or reverse effect sizes. Read codes beginning with ‘X00’, ‘XaE’ and ‘2BB’ were removed as they did not reach statistical significance with one or more AMD risk factors and had generally effect sizes close to zero (Table S1). We also excluded participants without data from general practitioners (i.e., around 60% of the UK Biobank, Figure S1).

**FIGURE 1:**
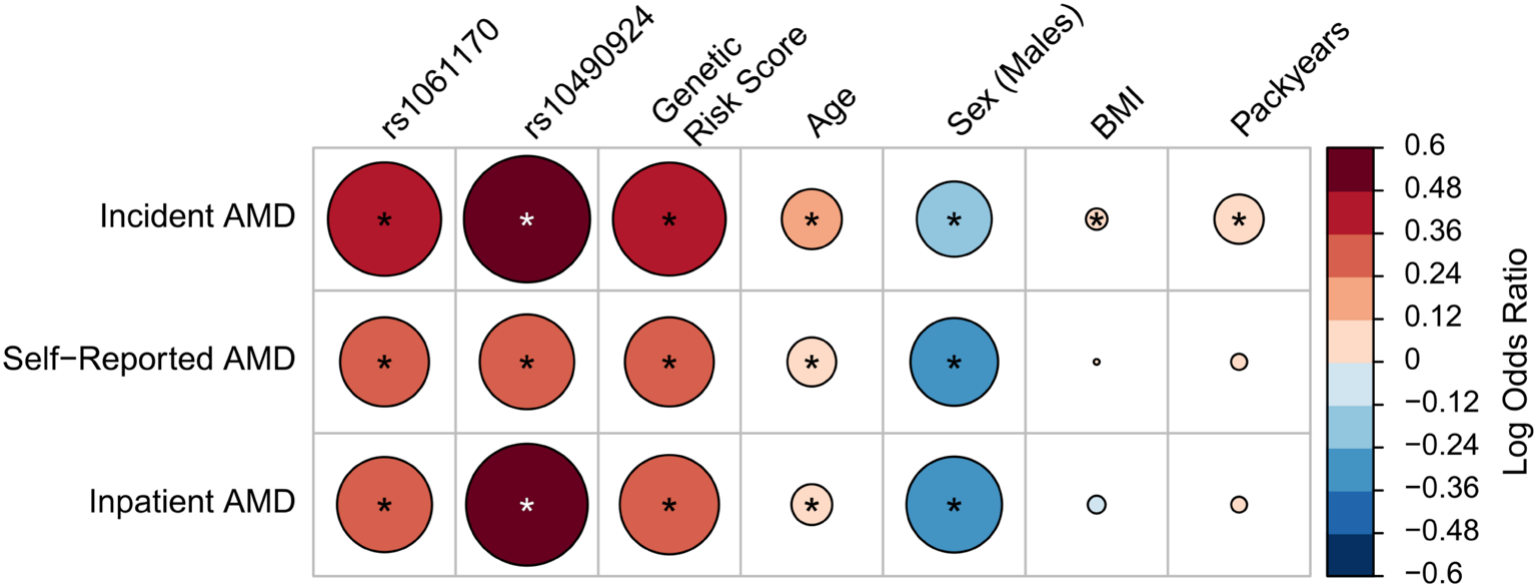
Association of known modifiable and non‐modifiable risk factors with different AMD definitions. The size of the circles represents the log of the odds ratio of each association. Blue colour indicates a protective effect (i.e., negative log odds ratio), while red is risk increasing (advserse). An asterisk (*p* < 0.05) represents associations that reached statistical significance at *p* < 0.05. BMI, body mass index.

Furthermore, to examine incident cases over time, we excluded participants diagnosed below the age of 40, as those would not reach 50 years of age by the end of the follow‐up (which was in 2017). We also excluded participants with prevalent AMD (i.e., with AMD‐specific read codes occurring before recruitment) from our analysis to create a longitudinal representation of AMD in the UK Biobank. After applying the standard exclusion criteria in the UK Biobank as described above, we obtained 1309 incident cases from the health electronic records. In addition to this definition, we also explored additional ways to identify AMD patients: The hospital episode data (HES) contains AMD diagnoses recorded by physicians during hospitalisation, and self‐reported data were gathered via a questionnaire on the day of recruitment. With those methods, we ascertained 3064 self‐reported prevalent AMD cases from the self‐reported questionnaire on the recruitment day and 657 AMD cases from the hospital episode spell data (HES). Finally, we compared each group according to their risk profile of known AMD‐associated risk factors (Table 1, Figure 1).

**TABLE 1:** Description of different AMD groups in the UK Biobank after quality control.

Variable	Incident AMD	Self‐reported AMD	HES AMD
Number of cases	1309	3064	657
Mean chronological age (SD)	62.86 (5.61)	61 (6.35)	60 (6.53)
Males	559	1229	254
Females	750	1835	403
Mean BMI	27.99	27.4	27.2
Smoking status (current)	134	252	51
Mean packyears (SD)	12.02 (19.0)	9.5 (17.72)	9.1 (16.14)

Abbreviation: SD, standard deviation.

### 2.4 Biological Age Computation

We estimated the biological age of each organ system separately using *Keras* and *TensorFlow* libraries in the R programming language (Graphical Abstract). We trained our models utilising the A100 GPUs of the hypercluster ‘Maxwell’ at the University of Aberdeen. Initially, we pre‐processed our different system datasets in an appropriate numerical format for the Keras package. Following this, we removed positive AMD participants to avoid potential bias, and we also removed participants with unknown AMD status, resulting in 187,961 total participants used in training and testing. Then, we split our dataset into 147,721 participants for the training dataset and 40,240 participants for the testing dataset to test our models' robustness and efficiency in unseen data (Figure S1). Using the R package *MatchIt*, we ensured our testing dataset median matched our AMD‐positive participants in chronological age, smoking behaviour and BMI.

During training, we assigned chronological age‐derived weights to our participants, with participants further away from the mean chronological age receiving heavier weights to minimise issues with heteroskedasticity in the predicted age values (i.e., larger residuals to chronological age further from the mean age). Our models were constructed with 3 layers with 2000 units,1000 units, 250 units, and a final layer with one neuron, *relu* as activation, and 0.5 dropout levels in between each layer. We compiled the modesl with the *adam* optimiser and mean squared error (mse) loss. Finally, we trained the models for 2000 epochs, with a batch size of 100, validation split of 0.2 (i.e., 20% of the training data was used to show the progress of the training in a validation dataset), and patience of 1000 epochs (i.e., training was stopped if the mse in the validation dataset did not improve for 1000 epochs). Furthermore, when the early stopping point was reached, we restored the weights of the best‐performing model according to the validation split. Finally, the coefficient of correlation between actual and predicted age was calculated for both training and testing datasets for comparison of the predictive power of the model. To construct the variables used in association testing, we computed the difference between predicted (biological) and chronological age in each organ system for each individual (age gap, Graphical Abstract). This is equivalent to computing the residuals in a regression between the predictors and the chronological age.

### 2.5 Genetic Risk Score Computation

The overall genetic risk score for AMD was computed by counting the number of risk‐increasing alleles, weighted (multiplied) by the log odds ratio of 48 known risk variants (Fritsche et al. 2016) (originally, 52 variants, 4 variants were not imputed in our UKB genotype dataset) with plink2 (Chang et al. 2015), as previously described (Grassmann et al. 2012). We also computed risk scores for AMD separately by the relevant molecular pathway of the underlying variants (Biarnés et al. 2020). In particular, we calculated a score representing variants found in or near genes involved in the function of the complement cascade, genes involved in lipid metabolism, and extracellular matrix and the remaining risk variants as a separate score (Table S2). Similarly, we computed the genetic risk score for IEAA (Intrinsic Epigenetic Age Acceleration), Hannum Age Acceleration and Pheno Age Acceleration epigenetic clocks using previously reported genetic risk variants. In total, 24 variants were used for IEAA, 9 for Hannum Age Acceleration and 12 for Pheno Age Acceleration (McCartney et al. 2021) (Table S3).

### 2.6 Statistical Analysis

We fit multivariable logistic models to assess the association of the incident AMD from the GP data, self‐reported AMD from the questionnaire on the day of recruitment, and in‐patient AMD from the HES with known modifiable and non‐modifiable risk factors of AMD jointly in R. To be more specific, we computed the association with age at baseline, sex, BMI, packyears, *CFH* variant rs1061170, *ARMS2* variant rs10490924, and a genetic risk score summarising the risk of all known AMD risk variants per person. The analyses were additionally adjusted for the first 10 principal components of ancestry. Furthermore, multivariable linear models were used to assess the association between biological ageing (represented as the difference between machine learning predicted and chronological age) with incident AMD (primary exposure), effectively yielding the biological age difference in years between cases and controls. Similarly, the correlation between biological ageing and the overall genetic risk score of AMD, the genetic risk scores of three different epigenetic clocks and lifestyle factors were evaluated. Those models were adjusted for age at baseline (to account for any residual effects of age in the analyses), the first 10 principal components of ancestry, packyears, alcohol and BMI to avoid potential confounding effects. Finally, we fit a multivariable linear regression to assess the association of the difference of biological and chronological age with AMD incident cases (main exposure), adjusted for all the above covariates plus the difference of chronological age and biological age of the other 6 organ system. We considered two‐sided *p*‐values below 0.05 as statistically significant.

## 3 Results

### 3.1 Assessing AMD Disease Status in the UK Biobank

Previous studies ascertained AMD disease status in the UK Biobank either by questionnaire at baseline or hospital episode spell data (inpatient hospital records). Inpatient AMD status includes only a small number of individuals who have been hospitalised for AMD (either before or after recruitment), making it inefficient for statistical analysis. Self‐reported AMD status is likely influenced by recall bias and generally does not correlate well with actual disease status, particularly for less advanced forms (McGuinness et al. 2024). Self‐reporting of AMD status also can only ascertain prevalent AMD cases, which is not ideal when the aim is to identify unbiased risk factors.

To overcome those limitations, we used the electronic health records of general practitioners (GP) data up until 2017. First, we extracted read codes previously described as relevant to AMD (see Warwick et al. 2023). We then added further read codes that are potentially relevant to AMD (e.g., read codes coding for senile macula degeneration or related terms) to improve our sample size. Next, we excluded read codes that did not show an association with known genetic risk factors or lifestyle factors such as smoking (Table S1). Codes that showed the expected association pattern were kept, identifying individuals with an AMD diagnosis. From those individuals, we removed prevalent cases (i.e., with a relevant read code recorded before recruitment), resulting in incident AMD cases only, reducing bias in the association tests. We then compared the three types of AMD definition (incident AMD from medical records, self‐reported and HES) with known modifiable and non‐modifiable risk factors (Table 1, Figure 1).

To this end, we assessed the association of incident AMD with *CFH* variant rs1061170 (Y402H), *ARMS2* variant rs10490924 (A69S), and the genetic risk score for AMD (computed from 48 risk variants in 34 loci as reported in Fritsche et al. (2016)). We found that incident AMD cases (OR: 1.59 [97.5% CI: 1.46;1.73], *p* = 1.37*10^−27^) showed a stronger association with the *CFH* variant rs1061170 than self‐reported or HES AMD. Furthermore, incident AMD cases were significantly associated with both the *ARMS2* variant rs10490924 (OR: 1.78 [97.5% CI:1.46; 1.73], *p* = 1.1*10^−36^) and the genetic risk score of AMD (OR: 1.58 [97.5% CI: 1.49; 1.68], *p* = 7.8*10^−53^). (Figure 1; Table S4). In addition, older age was shown to be a significant risk factor for incident AMD (OR: 1.14 [97.5% CI:1.12; 1.15], *p* = 3*10^−147^) as well as BMI and packyears (i.e., the number of years smoking one pack a day).

The self‐reported AMD status and the HES‐derived AMD status were also found to be associated with age but not with BMI and packyears (Figure 1; Table S4). In addition, those definitions showed a weaker association with the AMD genetic risk score. Thus, our analyses focused on using the AMD status derived from electronic health records (GP data, Figure S1).

### 3.2 Biological Age Prediction

Building on those results, we next computed participants' biological age in the UK Biobank to represent their organ‐specific biological age. This approach was done in individuals with available GP data (i.e., 189,270 individuals passing quality control). From this cohort, we used 147,721 non‐AMD individuals as the training dataset and 40,240 non‐AMD individuals as the testing dataset, which were median matched (selected) to incident AMD patients according to age, BMI and smoking status (Table 2).

**TABLE 2:** The datasets used for model generation and association testing.

Variable	Training set	Test set	Incident AMD
Mean chronological age (SD)	55.1 (7.73)	62.76 (5.72)	62.86 (5.61)
Number of individuals (age < 60) (%)	71,178 (48.18%)	19,393 (48.19%)	967 (73.87%)
Mean predicted age (excluding Hepatic) (SD)	55.1 (2.36)	56.5 (1.88)	56.74 (1.86)
Mean predicted age (Cardiovascular) (SD)	55.04 (3.44)	56.6 (3.47)	56.68 (3.46)
Mean predicted age (Pulmonary) (SD)	54.9 (3.72)	56.5 (3.27)	56.73 (3.28)
Mean predicted age (Musculoskeletal) (SD)	55.1 (3.6)	56.5 (3.06)	56.69 (2.99)
Mean predicted age (Metabolic) (SD)	55.1 (3.39)	56.3 (2.99)	56.50 (3.06)
Mean predicted age (Immune) (SD)	55.1 (3.39)	56.3 (2.99)	56.63 (2.89)
Mean predicted age (Hepatic) (SD)	55.09 (3.31)	56.1 (2.79)	55.97 (2.85)
Mean predicted age (Renal) (SD)	55.1 (4.25)	57.1 (3.67)	57.22 (3.69)
Number of individuals	147,721	40,240	1309
Males %	46.59%	42.09%	43%
Females %	53.41%	57.91%	57%
Mean smoking packyears (SD)	8.1 (15.34)	9.7 (17.69)	12.02 (19.09)
Missing packyears %	14.5%	15.7%	14.1
Mean genetic risk score (SD)	0 (0.02)	0 (0.02)	0.01 (0.02)

Abbreviation: SD, standard deviation.

Figure S2 shows the efficiency of each model in predicting the biological age in the training and testing dataset, respectively. The correlation coefficient (r) between predicted and chronological age for the hepatic system was 0.27 in the testing dataset. Similarly, we found correlation coefficients of 0.35 for the musculoskeletal, 0.38 for the pulmonary, 0.36 for the cardiovascular, 0.30 for the immune, 0.39 for the renal and 0.30 for the metabolic system in the test set, indicating a good predictive power for the respective models, in line with previous studies (Tian et al. 2023) (Figure S2; Table S5).

### 3.3 Association of Biological Age With AMD

We then investigated the association between modifiable and non‐modifiable factors with the age gap of the seven organ systems in the test set. Previous studies reported significant associations between BMI, packyears of smoking and alcohol with older biological age in multiple organ systems (Tian et al. 2023). As expected, we found that packyears and higher BMI were associated with an older biological age across all investigated organ systems (Figure S3; Table S6). Furthermore, consuming alcohol more than three times a week is associated with an older biological age of the metabolic system and a younger biological age of the renal, hepatic and pulmonary systems.

**FIGURE 2:**
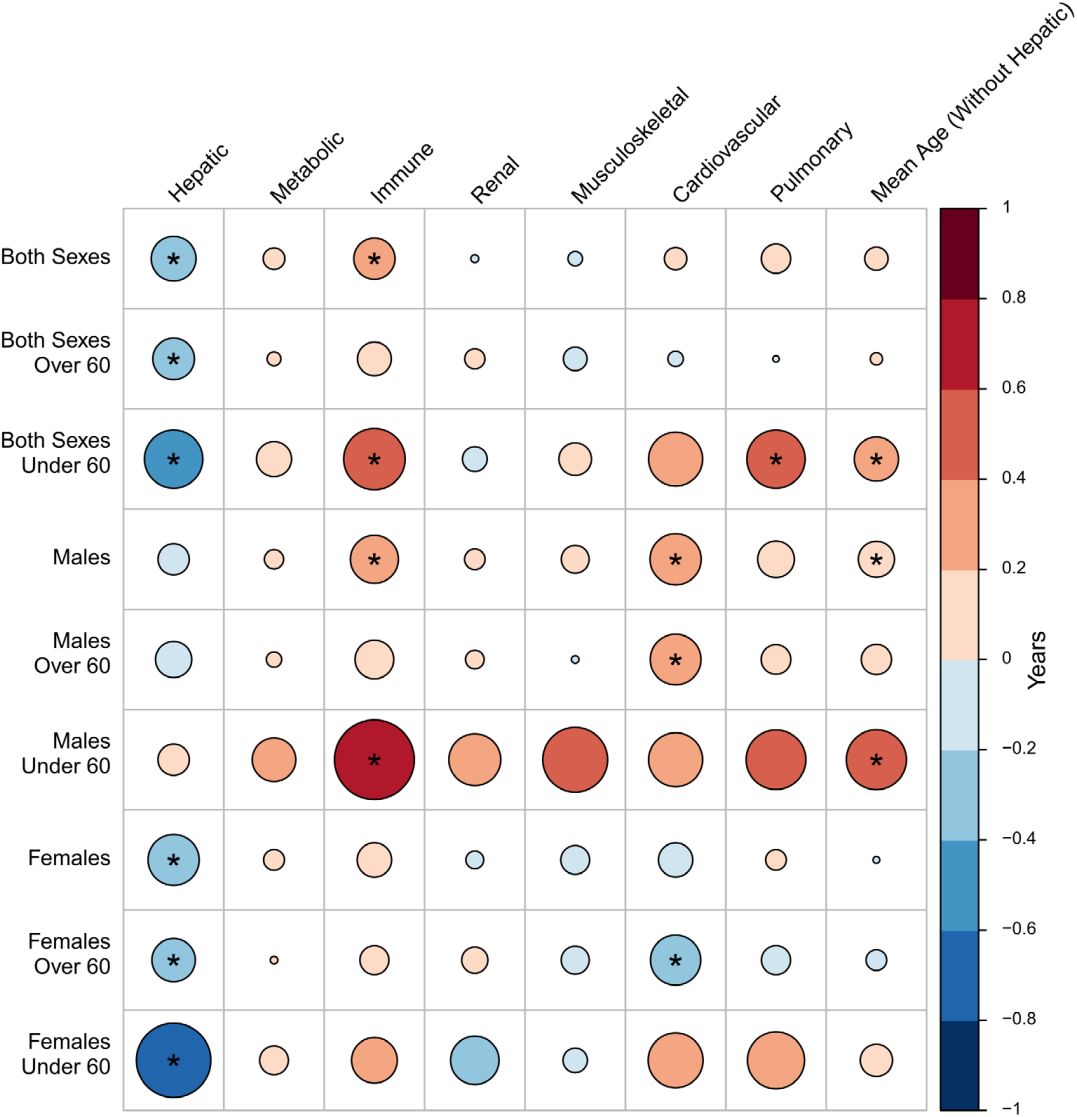
Association between biological ageing of different body systems and incident age‐related macular degeneration. The figure depicts the difference between chronological and biological age in AMD patients versus controls. Generally, AMD patients had accelerated organ ageing in all systems apart from the hepatic system. The circles' size represents the difference in years between chronological and biological age. Blue indicates a younger biological age of the mentioned organ system in AMD cases compared to controls, while red indicates the opposite. An asterisk symbolises a significant difference (*p* < 0.05).

Next, we computed the association of the age gap with incident AMD. In total, 16,937 male controls and 559 male incident AMD cases, as well as 23,306 female controls and 750 female incident AMD cases, were included in the analysis. We found a significant association between the increased biological age of the immune system and AMD (Figure 2). AMD patients showed significantly increased immune ageing compared to healthy participants (Difference: 0.21 years [97.5%CI: 0.06; 0.37], *p* = 6*10^−3^). Stratifying by chronological age, we noticed that the age difference in the biological age of the immune system in AMD patients and controls increased in the group below the age of 60 (Difference: 0.47 years [97.5%CI: 0.1; 0.85], *p* = 1*10^−2^). Similarly, male AMD patients showed increased immune ageing compared to male controls (Difference: 0.29 years [97.5%CI: 0.05; 0.53], *p* = 1*10^−2^), which increased in males under the age of 60 (Difference: 0.78 years [97.5%CI: 0.19; 1.36], *p* = 8*10^−3^). Although females with AMD also showed increased biological ageing, they did not reach statistical significance (Figure 2; Table S7).

Furthermore, our analysis revealed significant associations between the biological age of the cardiovascular system with AMD. Male AMD patients showed significantly increased biological ageing compared to healthy male participants (Difference: 0.3 years [97.5%CI: 0.05; 0.56], *p* = 1*10^−2^, Figure 2; Table S7). The difference remained similar and significant in the group above 60 years old (Difference: 0.3 [97.5%CI: 0.008; 0.59], *p* = 4*10^−2^), but it did not reach statistical significance in the group younger than 60. Interestingly, female AMD patients above the age of 60 showed decreased biological ageing compared to healthy participants' cardiovascular systems (Difference: −0.29 years [97.5%CI: −0.59; −0.009], *p* = 4*10^−2^).

No statistically significant associations were observed for the pulmonary metabolic, renal, or musculoskeletal ageing systems.

Of note, we observed that AMD patients (including both males and females) showed a decreased biological ageing of their hepatic system compared to controls (Difference: −0.25 years [97.5%CI: −0.4; −0.1], *p* = 7*10^−4^). The difference between chronological and biological ageing was larger in a population below the age of 6 (Difference: −0.41 years [97.5%CI: −0.78; −0.04], *p* = 2*10^−2^), and slimmer in a population above 60 years old (Difference: −0.22 years [97.5%CI: −0.38; −0.05], *p* = 7*10^−3^). We did not observe a statistically significant difference in males, but female AMD patients showed decreased biological ageing of their hepatic system compared to healthy females (Difference: −0.32 years [97.5%CI: −0.52; −0.11], *p* = 2*10^−3^). Similar to the analysis with both sexes, the difference between chronological and biological ageing increased in a population below the age of 60 (Difference: −0.67 years [97.5%CI: −1.18; −0.17], *p* = 7*10^−3^), while decreased in a population above 60 years old (Difference: −0.23 [97.5%CI: −0.45; −0.007], *p* = 4*10^−2^) (Figure 2; Table S7).

In addition, we performed a multivariable analysis to examine if there were any interactions between the biological age of different organ systems. To this end, the association of each organ ageing markers with incident AMD was assessed with a linear regression model, additionally adjusted for all remaining organ ageing markers. Generally, the observed associations detailed above remained similar with only minor deviations (Figure S4; Table S8), indicating that there is little covariation in the ageing parameters influencing our association results.

### 3.4 Genetic Factors of Biological Ageing

To better understand our findings, we investigated whether genetic risk variants of AMD are associated with increased or decreased biological ageing. We calculated an overall genetic risk score (GRS) based on 48 genetic variants across 34 loci. Following, we scaled the GRS in order the effect of GRS on biological age difference to be expressed in units of standard deviations (SD) of the GRS. We used data from 216,326 individuals with unknown AMD status who were not included in our training or testing datasets due to not having GP data available. Our analysis revealed significant associations between higher GRS values and increased biological ageing in several systems.

**FIGURE 3:**
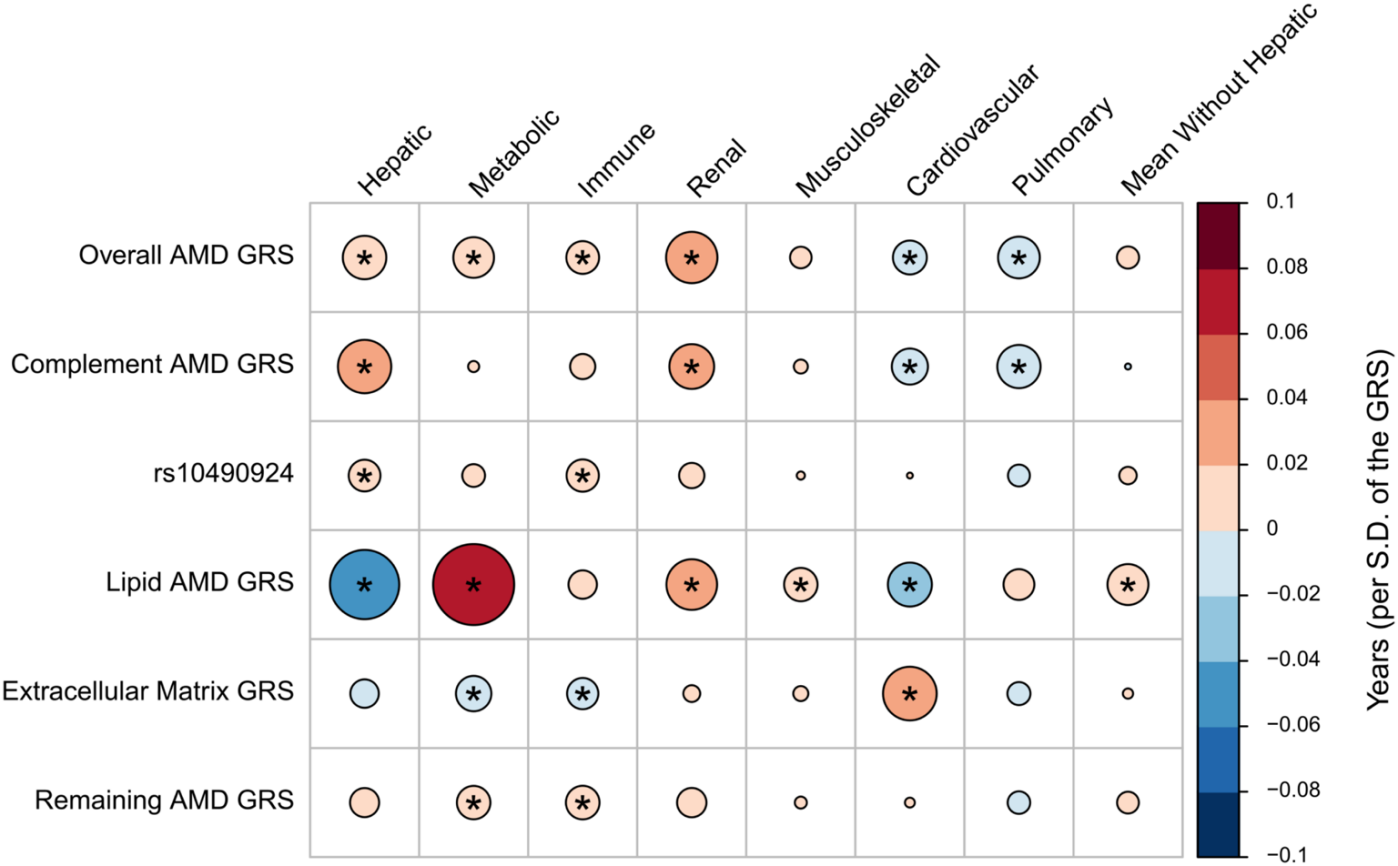
Associations between genetic risk factors of age‐related macular degeneration and biological ageing of several organ systems. Depicted is the correlation between genetic risk factors of AMD such as the overall genetic risk score (GRS, based on 48 AMD risk variants) as well as pathway specific GRS from variants located in the complement system, lipid metabolism, extracellular matrix, and rs10490924 in *ARMS2*. The circles' size and colour represent the slope for one standard deviation (SD) increase in genetic risk score. Blue indicates a protective effect against increased biological ageing (i.e., a higher score means lower biological age), while red is the opposite. An asterisk represents significant associations.

In particular, we observed that the overall GRS was significantly associated with increased biological ageing of the hepatic (Difference per SD of the GRS: 0.019 years [97.5% CI: 0.01; 0.029], *p* = 5*10^−9^), of the immune (Difference per SD of the GRS: 0.01 years [97.5% CI: 0.002; 0.021], *p* = 2*10^−2^), renal (Difference per SD of the GRS: 0.027 years [97.5% CI:0.016; 0.039], *p* = 2*10^−6^) and metabolic systems (Difference per SD of the GRS: 0.017 years [97.5% CI:0.007; 0.026], *p* = 7*10^−3^). On the other hand, the overall GRS was also significantly associated with decreased biological ageing in the pulmonary (Difference per SD of the GRS: −0.018 years [97.5% CI:‐0.029; −0.008], *p* = 6*10^−4^) and the cardiovascular system (Difference per SD of the GRS: −0.012 [97.5% CI:‐0.022; −0.002], *p* = 2*10^−2^) (Figure 3; Table S9).

Next, we separated our AMD risk variants depending on their putative pathways. We pooled them into genetic risk scores related to the complement system, the lipid metabolism system, the extracellular matrix, and the variant rs10490924 located in the *ARMS2* gene and the remaining variants as a separate group (Figure 3; Table S9). Interestingly, we found a more granular association pattern compared to the overall GRS of AMD. Specifically, the complement‐related AMD genetic risk score behaved similarly to the overall GRS. On the other hand, the variant rs10490924 was associated with accelerated biological ageing of the hepatic system (Difference per risk allele: 0.01 years [97.5% CI:0; 0.02], *p* = 4*10^−2^) and immune systems (Difference per risk allele: 0.01 years [97.5% CI:0.001; 0.02], *p* = 2*10^−2^). In contrast to the overall AMD score, the extracellular‐matrix AMD genetic risk score was significantly associated with increased biological ageing of the cardiovascular system and significantly associated with decreased biological ageing of the metabolic system and the immune system (Figure 3; Table S9). Similar to the overall score, the lipid‐related AMD genetic risk score was significantly associated with increased biological ageing of the metabolic, renal and musculoskeletal systems, as well as the mean biological age of all systems, excluding hepatic ageing. Intriguingly, the lipid GRS was significantly associated with decreased biological ageing with the cardiovascular and hepatic systems, potentially explaining part of the associations we saw for incident AMD risk (Figure 3; Table S9).

The majority of the observed associations retained their directions between the different sexes. However, we observed an inverted effect of the genetic risk score from variants in the lipid pathways between the biological age of males and females: a higher genetic risk score of lipid‐related variants was associated with a higher biological age of the immune system (Difference per SD of the GRS: 0.037 years [97.5% CI:0.023; 0.051], *p* = 1.16*10^−7^) in males and lower biological age of the immune system (Difference per SD of the GRS: −0.018 years [97.5% CI:‐0.031; −0.004], *p* = 1*10^−2^) in females (Figure S5; Table S9).

Finally, we studied the association of genetic risk scores of three previously established epigenetic clocks (intrinsic epigenetic age acceleration—IEAA, Phenotypic age acceleration—PhenoAgeAccel and Hannum age acceleration—HannumAgeAccel) with incident AMD. However, we did not observe any significant associations at *p* < 0.05. Nevertheless, we found that the higher genetic risk score for the PhenoAgeAccel epigenetic score was associated with older immune, musculoskeletal and mean biological age (excluding the hepatic system) (Figure S6; Table S10). Similarly, the HannumAgeAccel was associated with older immune and pulmonary systems (Figure S6; Table S10). In contrast, the genetic risk score of IEAA was associated with younger metabolic and older musculoskeletal age.

## 4 Discussion

In this study, we first ascertained incident AMD cases from electronic health records by identifying AMD‐relevant disease codes that are significantly associated with genetic and non‐genetic AMD risk factors. Next, we report that incident AMD cases show a differential biological ageing pattern across various organ systems compared to individuals who did not develop AMD. While AMD patients generally had an increased biological ageing compared to controls, we found the opposite to be true for the biological ageing of the liver. The observed association pattern can partially be explained by genetic risk variants in or around genes in specific pathways, which will be crucial to dissect in the future.

Previous studies have underlined the concept of biological ageing and shown the importance of differential ageing of different physiological systems and organs (Ferrucci et al. 2020; Tian et al. 2023; Tuttle et al. 2020) on disease risk and survival. However, the role of differential organ ageing in AMD risk and its relation to the strong genetic risk factors has not yet been explored. We assessed the association of the age difference between the chronological and biological age of those systems with incident AMD cases, stratifying out analyses by sex and age. To ensure other confounders did not influence our analyses, we adjusted for important lifestyle factors, like smoking, BMI, alcohol consumption and potential population differences, by including ancestry principal components 1–10. Apart from those factors, only a few risk factors are remaining that are known to be involved in AMD risk (such as CRP levels, sunlight exposure, or blood lipid levels). In addition, we are limited to adjusting for factors that were not used to model biological ageing, as including those factors in the association testing would introduce co‐linearity, which could violate some of the assumptions of the models. As such, accounting for CRP levels, high‐density lipoprotein measurements (HDL) and vitamin D levels (as a proxy for sunlight exposure) were not possible. However, we suppose that the adjustments laid out above should approximate even unaccounted factors due to their correlation to those confounders. Thus, it is unlikely that other large confounders will influence our analyses and that we, therefore, accounted for the major environmental/lifestyle factors involved in AMD.

In our analyses, we also investigated genetic risk scores for faster ageing according to several epigenetic genetic clocks with at least five variants that reached genome‐wide significance (McCartney et al. 2021). However, none of the genetic risk scores showed a significant association with incident AMD in our study. This can, however, also be an issue of power as we only included around 1309 individuals with incident AMD. Since the genetic risk is present since birth, such analyses could potentially be done in a larger cohort of samples with prevalent AMD or with a different technique such as Mendelian Randomisation. Nevertheless, we observed that the different genetic risk scores for epigenetic ageing also show a differential correlation with biological ageing. The statistically significant correlations generally point to older biological age compared to chronological age. However, the genetic score for faster intrinsic epigenetic age acceleration was associated with lower metabolic ageing. This would be in line with previous reports linking IEAA with lipoprotein levels, which were also used to model ageing in our cohort (Lu et al. 2018). It is important to note, that we did not directly assess the association of IEAA (i.e., the genetic score capturing part of the IEAA) with lipoprotein markers but the part of the markers that jointly model ageing. Therefore, additional analyses are warranted to understand the interplay between the genetically predicted epigenetic clocks and organ ageing.

The immune system of AMD patients showed faster ageing compared to controls, with younger than 60‐year‐old males exhibiting even faster ageing of their immune system compared with older males. The observed results are not surprising given a large portion of the disease risk for AMD can be explained by chronic exposure to increased complement activation (Paun et al. 2016; Weber et al. 2014). Nevertheless, we did not observe a direct link between genetic risk for AMD in complement genes and accelerated ageing of the immune system. However, the major AMD risk variant rs10490924 and the remaining variants increased the age difference between biological and chronological immune age and could partially explained the observed association. Although we adjusted for known environmental and other lifestyle factors in the analyses and focused on incident AMD cases, residual confounding could still occur and explain the difference in immune ageing between controls and AMD patients. Alternatively, unknown AMD risk factors not covered in our analyses, such as diet, infections, medication and cholesterol levels, could lead to accelerated immune ageing. Otherwise, the ageing‐associated component of the investigated immune markers might not be strongly correlated to complement activation, which would require further studies to untangle.

We showed that AMD patients had a younger biological age of their hepatic system compared to controls. Stratifying the analysis by sex, we found that predominantly female AMD patients had a younger biological age of their hepatic system since males did not reach statistical significance. The hepatic system's protection against faster biological ageing in AMD patients is not easily explained. However, we identified AMD risk variants located in genes associated with lipid metabolism that had a protective effect against accelerated ageing of the hepatic system, particularly in women. Variants protecting against faster biological age of the hepatic system and increased risk of developing AMD could be evolutionary trade‐offs, explaining the increasing prevalence of AMD and the puzzling large effect sizes on AMD risk observed for those variants. In contrast, the genetic risk score of complement‐associated variants resulted in increased hepatic ageing. This could be due to increased production of complement system components or due to stress of the hepatic system caused by misfolded complement proteins due to genetic variants associated with AMD. However, the overall genetic score for AMD towards increased hepatic ageing with higher AMD risk scores. Other biological explanations could be non‐genetic, like sun exposure. Sun exposure is considered a risk factor for AMD despite its vital role in producing vitamin D3, which protects the hepatic system (Keane et al. 2018). Interestingly, vitamin D supplementation was not successful in preventing AMD (Christen et al. 2020).

Generally, AMD risk variants (as measured by the various genetic risk scores) increase renal ageing. This is particularly the case for variants near genes related to complement cascade and lipid metabolism. It has been shown that the rare variant rs121913059 in the *CFH* gene strongly increases the risk for AMD and also for the atypical haemolytic uremic syndrome (Martinez‐Barricarte et al. 2008), highlighting a shared disease aetiology. Indeed, the aggregate complement genetic risk score could represent a significant complement pressure on the renal system, causing the observed accelerated ageing. The association between AMD risk variants located in genes related to lipid pathways and accelerated renal ageing is, however, more difficult to explain since lipids such as HDL showed a U‐shaped association with kidney disease (Nam et al. 2019). We can speculate that the changes in the lipid system caused by AMD‐associated variants could lead to increased systemic inflammation, which would increase kidney ageing (Ebert et al. 2020). The precise mechanism, however, requires further studies.

AMD risk variants located in or near genes associated with the complement system function and lipid pathways were protective against accelerated cardiovascular system ageing. Indeed, previous studies showed that AMD patients (including both males and females) are at genetically reduced risk for cardiovascular diseases (Grassmann et al. 2017b) and their pulmonary system is in better health (Grassmann et al. 2017b). Variants near lipid genes (such as *APOE*, *LIPC*, *LPL* and *CETP*) are known to influence total cholesterol, increase HDL as well as decrease LDL in the blood (Klarin et al. 2018; Zhou et al. 2013) and may also influence response to statins (Barber et al. 2010). Mendelian randomisation studies implicated a higher risk for AMD due to higher HDL (Burgess and Davey Smith 2017). Importantly, AMD patients seem to have higher levels of HDL in their blood compared to controls (Colijn et al. 2019), which could be partially responsible for their protection against cardiovascular disease. The above observations align with our findings, showing females over 60 with AMD having slower ageing of their cardiovascular systems compared to females over 60 without AMD. Interestingly, males diagnosed with AMD showed faster ageing of their cardiovascular systems compared to males without AMD. The difference could be attributed to variants not associated with AMD or environmental and lifestyle factors.

In contrast to the genetic risk score based on variants in lipid pathways, a genetic score representing the genetic risk conferred by the extracellular matrix (ECM) homeostasis (including variants near *TIMP3*, *VEGFA* and collagen genes) was found to affect ageing in the cardiovascular system in the opposite direction. TIMP3 is increased in several cardiovascular diseases (Mukherjee et al. 2006; Polyakova et al. 2011), while uncontrollable expression of VEGF‐A (similar to neovascular AMD) has been linked to higher risk and worsened severity of cardiovascular diseases (Cho et al. 2006; Meng et al. 2009). Further research is, however, necessary to identify the variants underlying those associations. In addition, we grouped the variants according to the function of their closest gene. This approach could wrongly assign variants to a certain pathway. Instead, the scores could be weighted further by incorporating gene expression results and/or effect sizes from other associations such as the correlation between the variant and lipid levels. This would allow a better view of the underlying biology involved in the association of those scores.

Currently, the genetic risk variants identified so far explain a sizable fraction of the disease risk for AMD (up to half for the overall risk score for AMD). Nevertheless, it is important to note that the effects observed for the genetic risk scores on biological ageing are relatively small (i.e., less than one month per standard deviation of the score). Hence, non‐genetic factors are likely more significant contributors to the observed effects on ageing than genetic components and genetic components can only provide partial insights into the underlying biology. However, the observed differences, even small, reveal differences between different body systems. Although a direct clinical significance of our findings is not obvious at this stage, we still provide novel pathophysiological insights in AMD and its genetics that future studies can leverage to focus on the immune, cardiovascular and hepatic systems to discover potential molecular pathways that can be targeted and have a meaningful clinical impact—especially the link between older immune and younger hepatic systems and incident AMD. Moreover, our study's findings, along with known AMD risk factors, could improve the prediction of AMD as well as prevention strategies by promoting lifestyle changes that positively influence the immune and cardiovascular systems. Finally, future studies should expand on other ethnicities to understand the effect of ancestry components in both biological ageing of the organ ageing and AMD.

In summary, we report that individuals who developed AMD showed accelerated ageing of their immune and cardiovascular systems and slower ageing of their hepatic system independent of potential and known confounders. Biological ageing is a complex process governed not only by lifestyle choices but also by genetic factors, but its predictive value could help in earlier diagnosis of age‐related macular degeneration and co‐morbidities in patients.

## Author Contributions

**Anastasios Papadam, Felix Grassmann:** conception and design. **Anastasios Papadam:** financial support. **Anastasios Papadam, Felix Grassmann, Arimantas Lionikas:** collection and assembly of data. **Felix Grassmann, Anastasios Papadam:** data analysis and interpretation. **Felix Grassmann, Anastasios Papadam:** manuscript writing. All authors: critical review of draft manuscripts and approval of final version.

## Conflicts of Interest

The authors declare no conflicts of interest.

## Supporting information


Figure S1.

Table S1.

## Data Availability

The UK Biobank Data are available upon request from the www.ukbiobank.ac.uk. The code to compute the AMD status in the UK Biobank and the analysis scripts will be published on the group's GitHub page: https://github.com/GrassmannLab.
