# Supplementary material for: Differential Organ Ageing Is Associated With Age‐Related Macular Degeneration
Source: Aging Cell. 2025 Jan 5;24(5):e14473. doi: 10.1111/acel.14473 (PMC12073918; doi:10.1111/acel.14473)
Supplement: Supplementary file 1 — Figure S1. [file ACEL-24-e14473-s001.docx]

**Supplementary Figures**


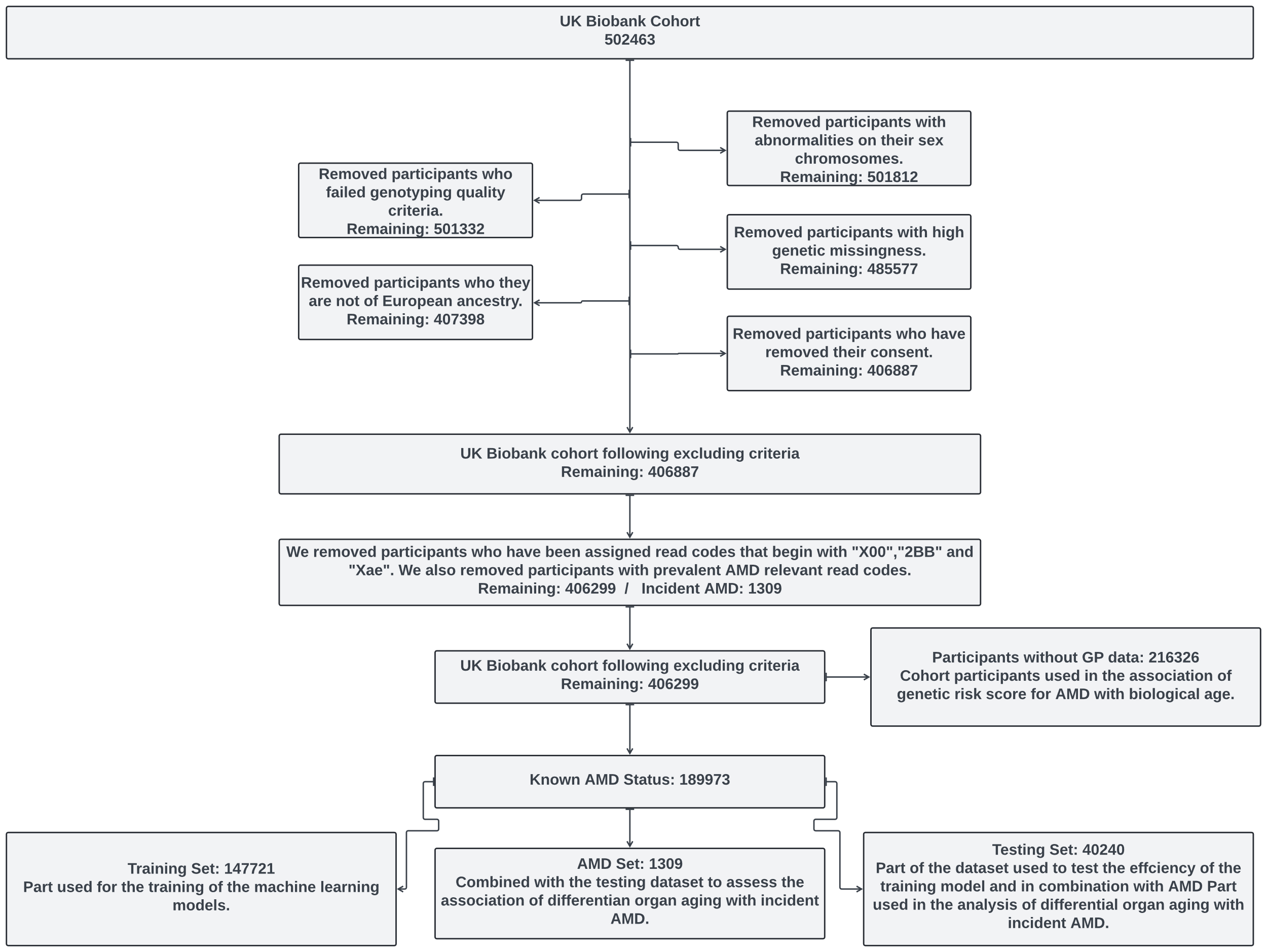


Supplementary Figure 1: A flowchart depicting the excluding criteria we applied, and the number of samples left after each step.


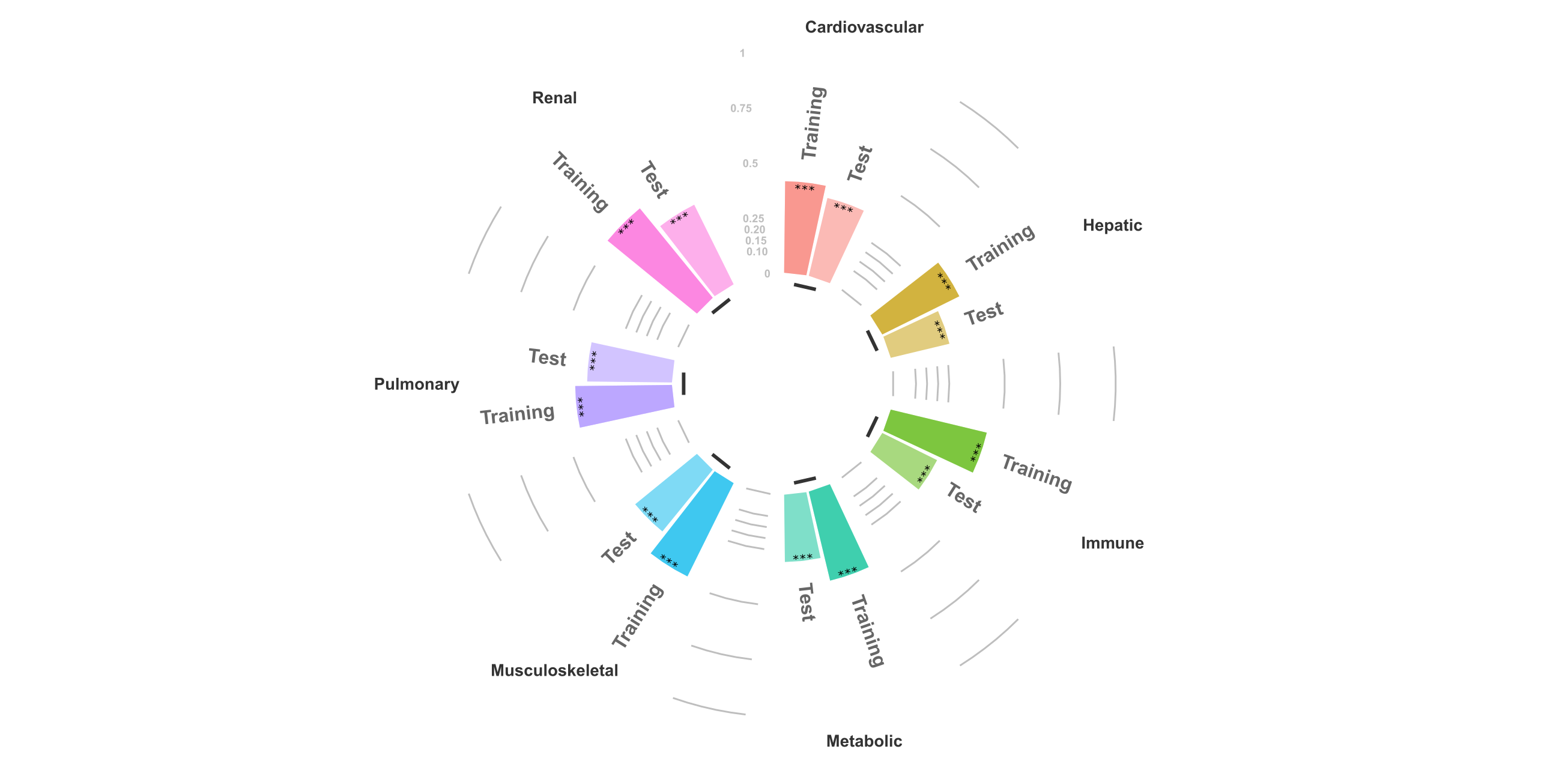


**Supplementary Figure 2: A circular plot depicting the coefficient correlation r for all seven organ systems used to assess the differential organ aging of participants in the UK Biobank**. The coefficient of correlation R is a statistical measurement of the data's proximity to the fitted regression line. As expected, the training datasets show higher R values than the testing datasets. The P values are depicted by asterisk with *** corresponding to P<0.000001. See **Supplementary Table 5** for more details.


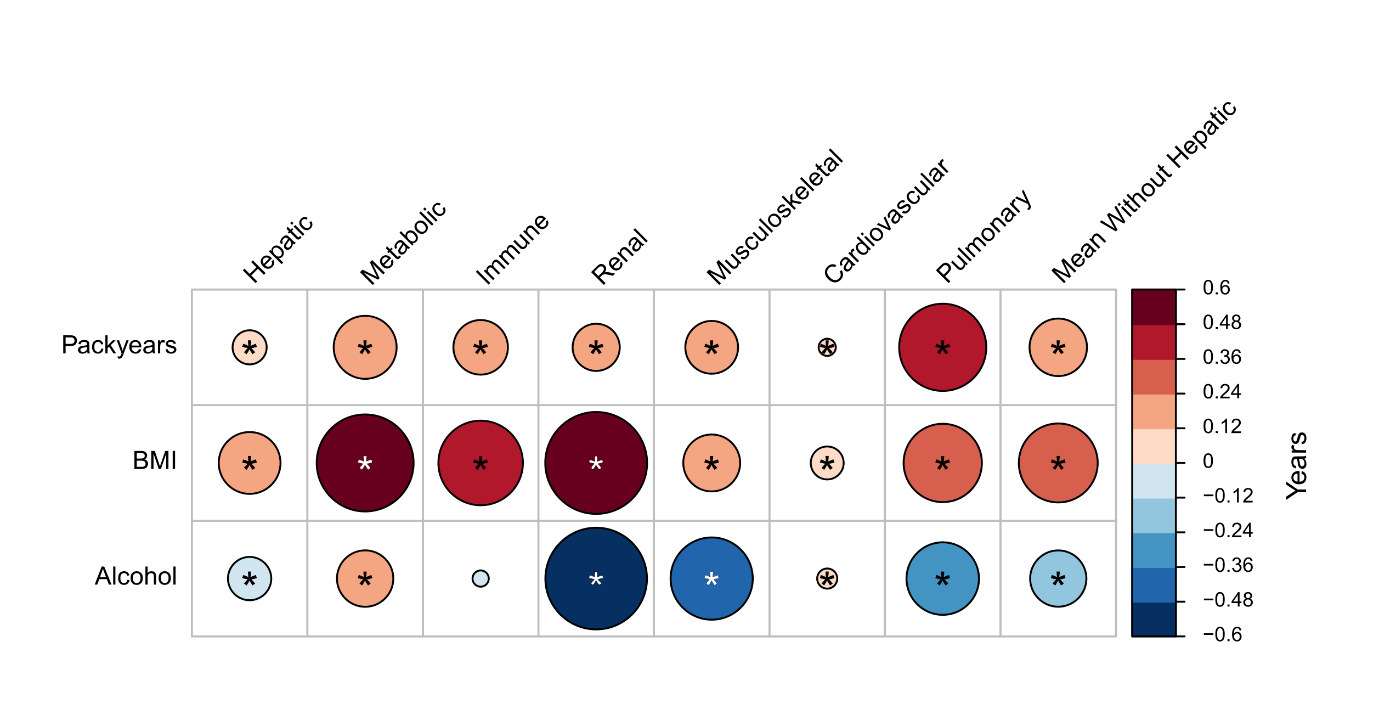


**Supplementary Figure 3: Associations between modifiable risk factors of age-related macular degeneration and biological** **aging of several organ systems:** Depicts the associations of modifiable lifestyle factors with biological aging of the hepatic, metabolic, immune, musculoskeletal, cardiovascular, pulmonary, and mean biological aging. The circles' size represents the slope of the regression with the expected change in the biological age in years of each system for each standard deviation increase of packyears and BMI; and if they drink more than 3 times a week. Blue indicates a protective effect against increased biological aging, while red is the opposite. An asterisk symbolizes a significant difference (P < 0.05).BMI = Body Mass Index


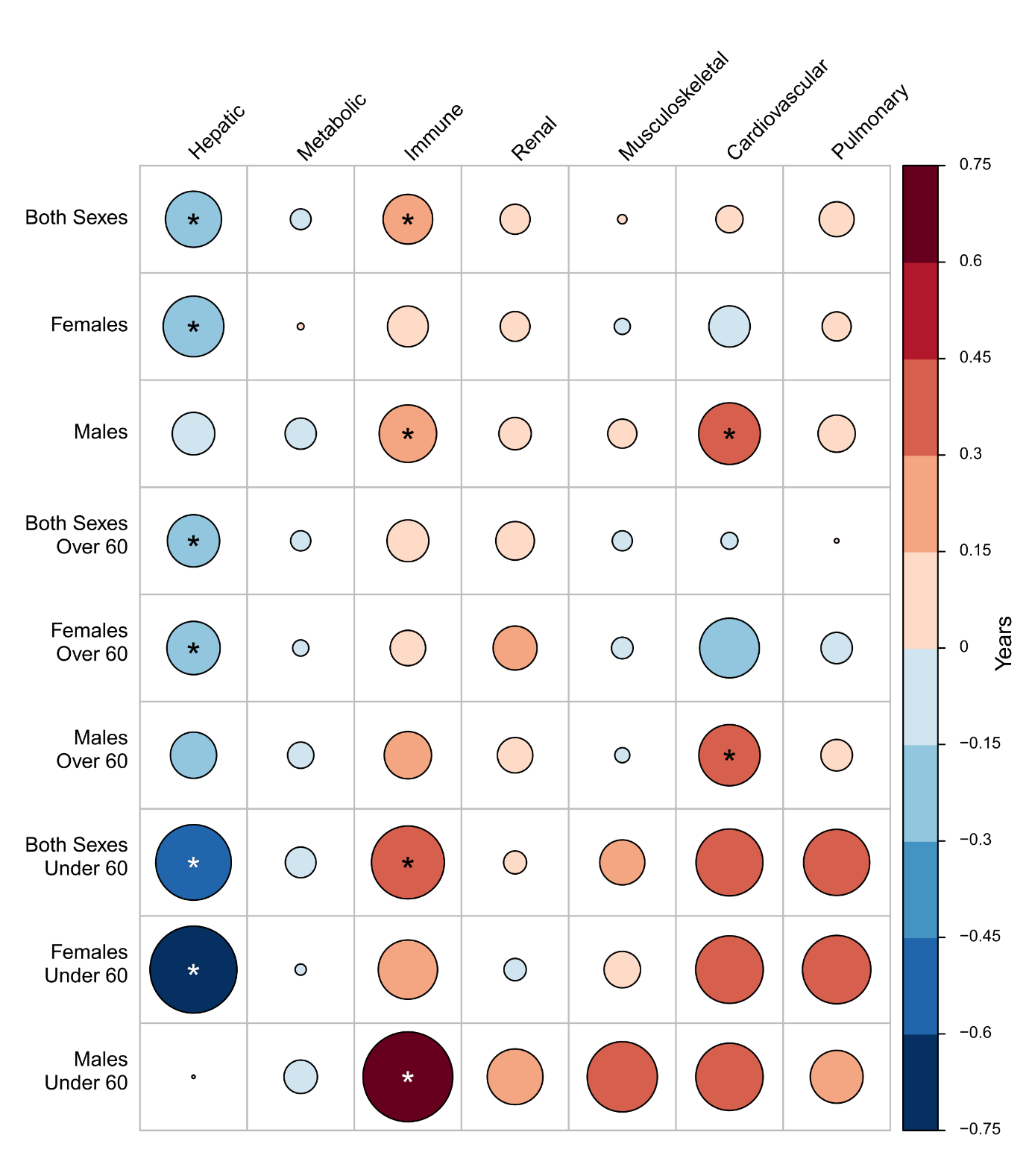


**Supplementary Figure 4: Multivariate association between biological aging of different body systems and incident age-related macular degeneration.** The circles' size represents the difference in years between chronological and biological age. Blue indicates a younger biological age of the mentioned organ system in AMD patients, while red indicates the opposite. The figure depicts the difference between chronological and biological age in AMD patients versus controls, revealing biologically older and younger organ systems in AMD patients compared to controls. An asterisk, which symbolizes a significant difference (P < 0.05). The analysis above is adjusted for lifestyle and environmental markers as well as the other organ systems' biological age.


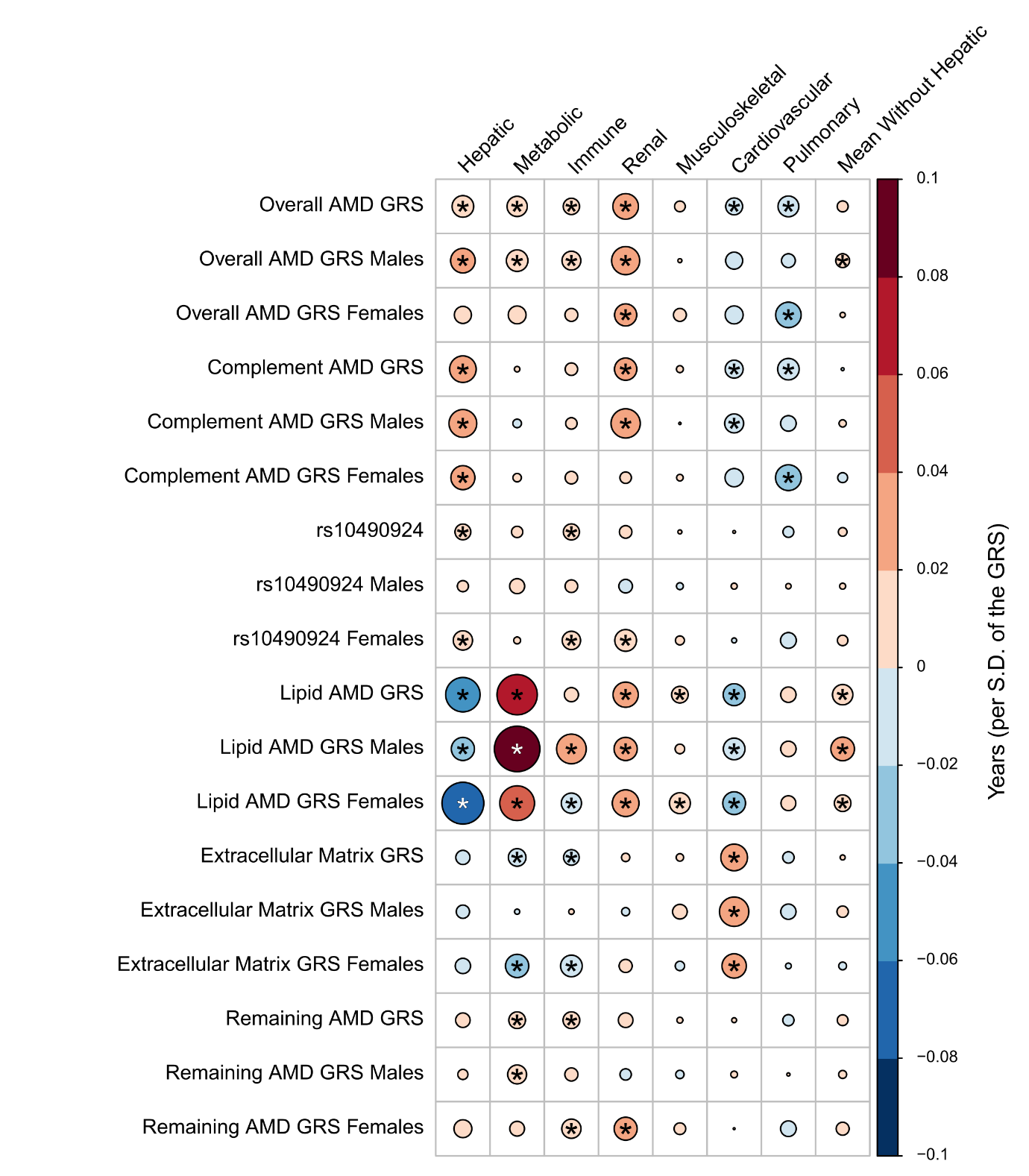


**Supplementary Figure 5: Sex-stratified associations between biological aging of several organ systems and genetic risk factors of age-related macular degeneration**: Depicts the associations of genetic risk factors of AMD with increased biological aging of the hepatic, metabolic, immune, musculoskeletal, cardiovascular, pulmonary, and mean biological aging. The circles' size represents the slope of the regression with the expected change in the biological age in years of each system for each standard deviation increase of the genetic risk score. Blue indicates a protective effect against increased biological aging, while red is the opposite. An asterisk symbolizes a significant difference (P < 0.05).


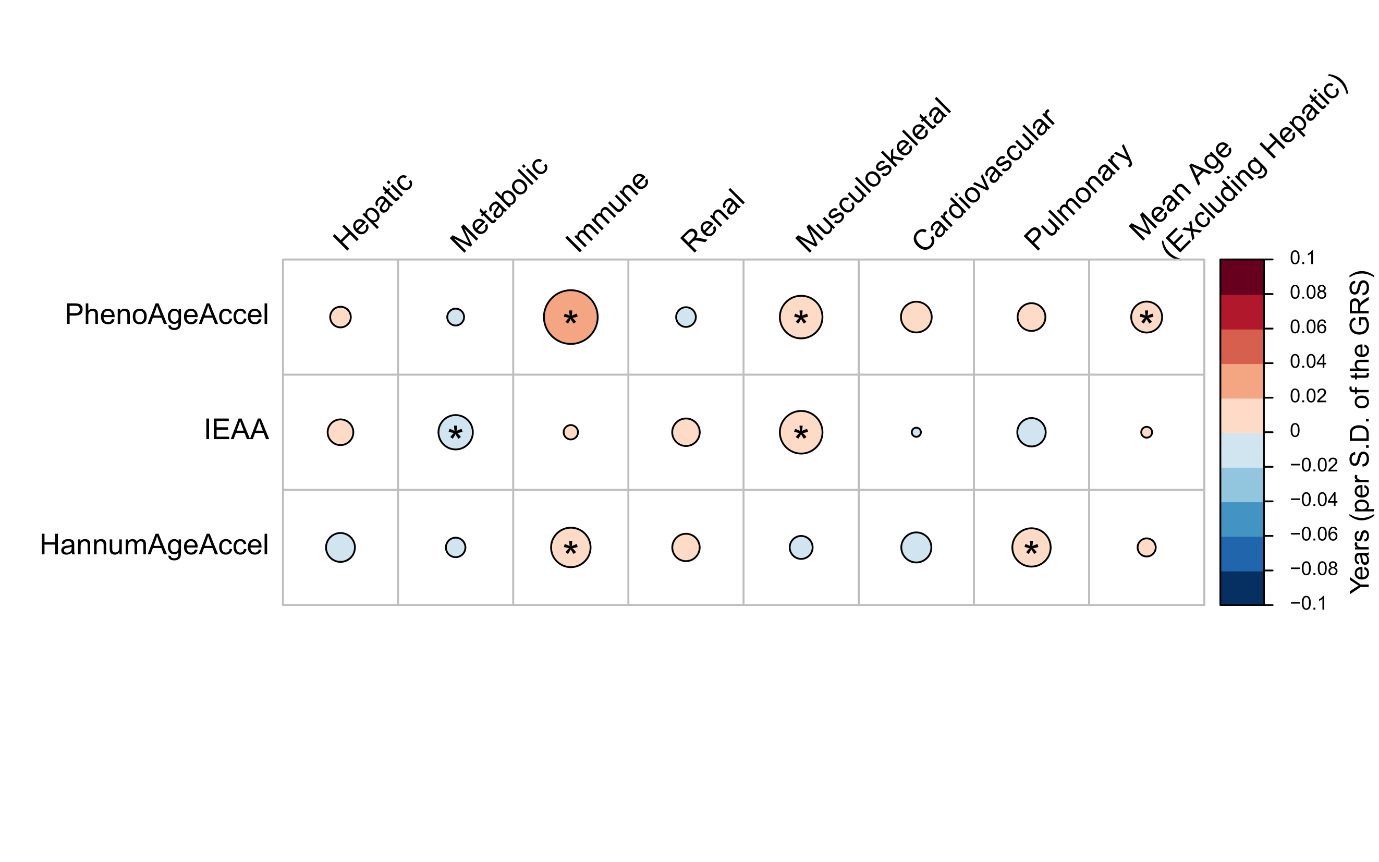


**Supplementary Figure 6: Correlation between genetic risk score of three different epigenetic clocks and chronological and biological age differences.** The circles' size represents the difference of years per one standard deviation (S.D.) of the genetic risk score (GRS). The red size shows that an increased GRS of the epigenetic clock results in a larger (more positive) gap between chronological and biological age (older biological age). Generally, the genetic risk scores of the epigenetic clocks are correlated with increased organ aging, apart from a younger metabolic age for the IEAA Score. An asterisk symbolizes a significant difference (P < 0.05). IEAA = Intrinsic Epigenetic Age Acceleration
